# Giant cardiac hemangioma: case report and literature review

**DOI:** 10.1093/ehjcr/ytag535

**Published:** 2026-10-06

**Authors:** Henrique Coutinho, Gustavo Kikuta, Marcos Filgueiras, Bruno Reznik Wajsbrot, Fabiana Resende Rodrigues

**Affiliations:** Department of Cardiovascular Surgery, Pedro Ernesto University Hospital, Boulevard 28 de Setembro, 77, Vila Isabel, Rio de Janeiro, RJ 20551-030, Brazil; Department of Cardiovascular Surgery, Serra dos Órgãos University Center (UNIFESO), Avenida Alberto Torres 111, Alto, Teresópolis, RJ 25964-004, Brazil; Department of Cardiovascular Surgery, Pedro Ernesto University Hospital, Boulevard 28 de Setembro, 77, Vila Isabel, Rio de Janeiro, RJ 20551-030, Brazil; Department of Cardiovascular Surgery, Serra dos Órgãos University Center (UNIFESO), Avenida Alberto Torres 111, Alto, Teresópolis, RJ 25964-004, Brazil; Department of General Surgery, Serra dos Órgãos University Center (UNIFESO), Avenida Alberto Torres 111, Alto, Teresópolis, RJ 25964-004, Brazil; Department of Cardiovascular Surgery, Pedro Ernesto University Hospital, Boulevard 28 de Setembro, 77, Vila Isabel, Rio de Janeiro, RJ 20551-030, Brazil; Department of Pathology, Antonio Pedro University Hospital, Rua Marquês do Paraná 303, Centro, Niterói, RJ 24033-900, Brazil

**Keywords:** Cardiac haemangioma, Primary cardiac tumour, Pericardial mass, Cardiac surgery, Multimodality imaging

## Abstract

**Introduction:**

Primary cardiac tumours are rare, with haemangiomas constituting only 2% of benign cardiac tumours. Although benign, these lesions can compress adjacent cardiac structures, leading to symptoms such as arrhythmias, heart failure, or even sudden death. This study presents a rare case of a giant cardiac haemangioma and provides a review of the current literature on its diagnosis and management.

**Case Report:**

A 58-year-old female presented with progressive abdominal pain and dyspnoea. Imaging revealed a large, heterogeneous mass in the pericardial sac measuring 11.9 × 11.7 × 6.7 cm. Complete surgical resection was performed without complications. Histopathological analysis confirmed the diagnosis of a cavernous haemangioma. Postoperative recovery was uneventful, and follow-up imaging showed no residual mass or functional impairment.

**Discussion:**

Cardiac haemangiomas are rare and pose significant diagnostic challenges. Advanced imaging modalities, such as magnetic resonance imaging (MRI) and computed tomography (CT), are essential for accurate diagnosis and surgical planning. Complete tumour resection remains the treatment of choice, ensuring a favourable prognosis and minimal risk of recurrence.

**Conclusion:**

Effective management of giant cardiac haemangiomas requires early diagnosis, precise imaging, and complete surgical resection. Long-term follow-up is necessary to monitor for recurrence and ensure optimal outcomes.

Learning points

- Giant cardiac haemangiomas may be incidentally detected despite large tumour size.
- Coronary angiography can identify feeding vessels and guide operative strategy.

## Introduction

Primary cardiac tumours are exceedingly rare, with a prevalence of 0.001–0.03% in routine autopsy series.^1,2^ Among them, cardiac haemangiomas are even less common, comprising approximately 2% of benign cardiac tumors.^2,3^ Despite their benign nature, these tumours can compress adjacent cardiac structures, leading to critical symptoms such as arrhythmias, heart failure, or sudden death, depending on their size and location.^4,5^

Histologically, cardiac haemangiomas are classified into three main subtypes: capillary, cavernous, and arteriovenous.^6,7^ The clinical relevance of these tumours lies not only in their potential to grow expansively but also in their ability to invade or compress cardiac structures, causing severe haemodynamic complications such as valve obstruction or impaired ventricular function.^5,8^

This article presents a rare case of a giant cavernous haemangioma located in the pericardial sac, accompanied by a review of the literature on the diagnostic challenges and therapeutic management of this condition.^9,10^

## Summary figure

**Figure:**
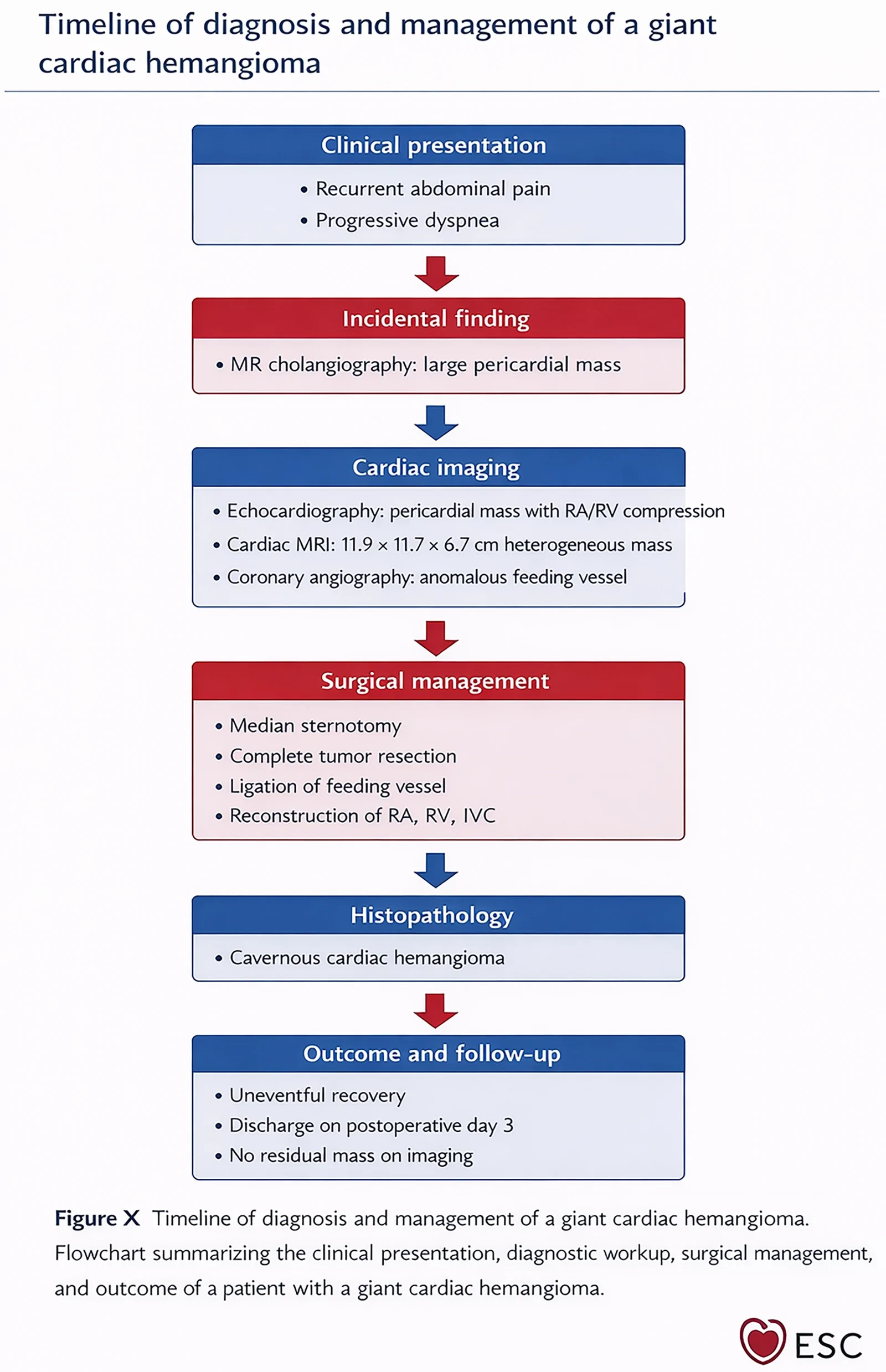


## Case report

A 58-year-old female patient presented with three episodes of severe abdominal pain and dyspnoea. With a history of cholecystectomy, she was initially investigated for choledocholithiasis, and magnetic resonance cholangiography revealed the presence of a bile duct stone, along with a large heterogeneous mass, with well-defined borders, located inside the pericardial sac and in close contact with the cardiac ventricular chambers, measuring 11.9 × 11.7 × 6.7 cm.

The patient was referred to the cardiac surgery department at the University Hospital of Rio de Janeiro State University. During hospitalization, baseline electrocardiography demonstrated normal sinus rhythm without conduction abnormalities, ischaemic changes, or arrhythmias. A bedside echocardiogram revealed normal-sized and normal-thickness cardiac chambers, an ejection fraction of 80%, normal valve morphology and function, and the presence of a homogeneous, iso-echoic mass adhered to the pericardium, measuring approximately 10 × 9 centimetres (*Figure 1*). The mass caused slight compression of the right atrium and right ventricle, without significant haemodynamic repercussions.

**Figure 1:**
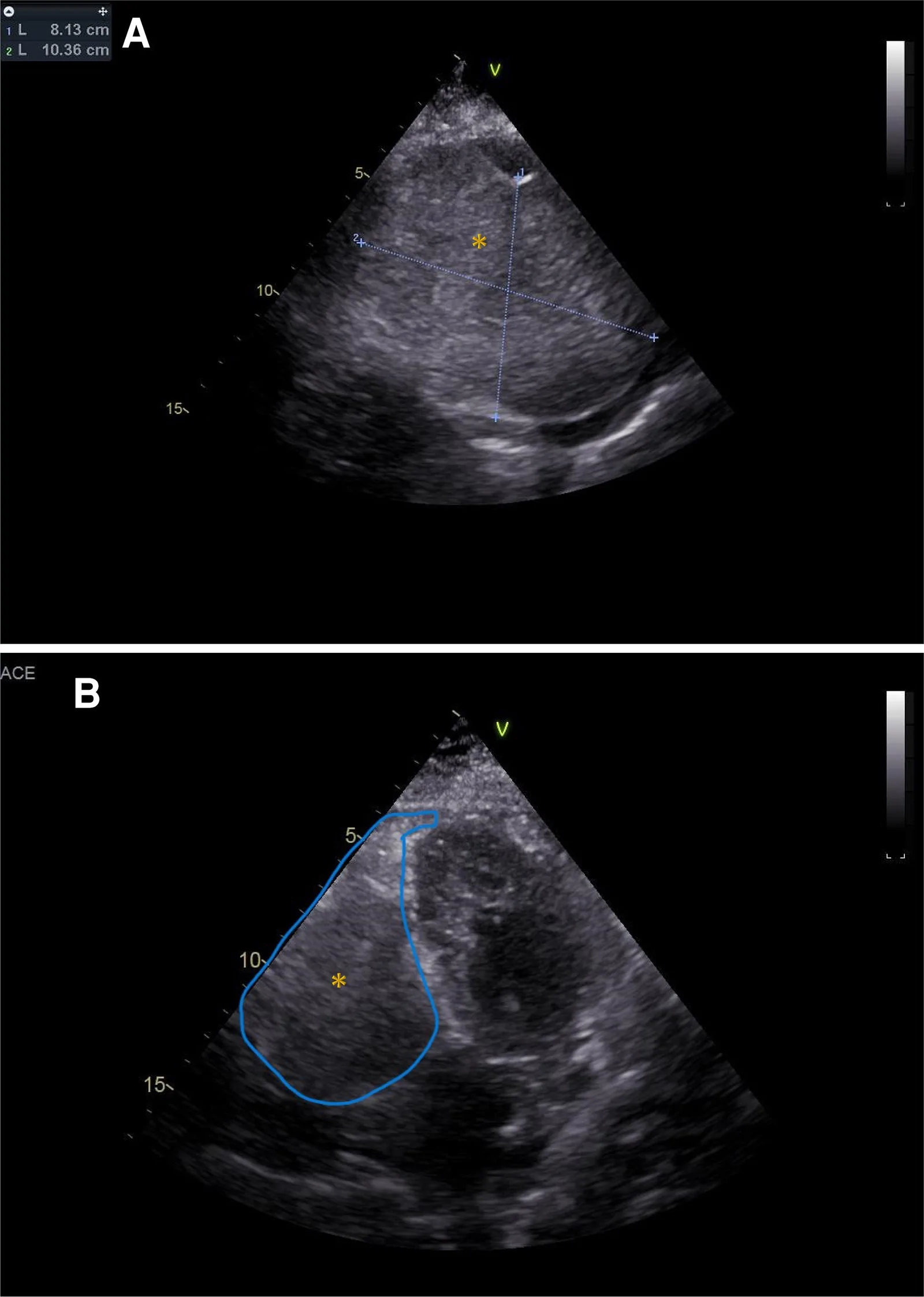
Transthoracic echocardiography showing an iso-echoic mass adhered to the pericardium and causing slight compression of the right cardiac chambers. (*A*) Apical four-chamber view demonstrating the mass. (*B*) Delineation of the lesion highlighting its relationship with the right cardiac chambers.

Cardiac magnetic resonance imaging (MRI) confirmed adequately sized cardiac chambers, without ventricular dysfunction and an ejection fraction of 62%. No valve abnormalities were identified. Moderate pericardial effusion was observed, predominantly in the inferior segments, but without signs of chamber restriction. A large pericardial mass with internal septations and necrotic areas, measuring 11.9 × 11.7 × 6.7 cm, was confirmed (*Figure 2*). The discrepancy between echocardiographic and MRI-derived ejection fraction values likely reflects methodological differences, as cardiac MRI volumetric assessment is generally more accurate and less operator-dependent than visual echocardiographic estimation.

**Figure 2:**
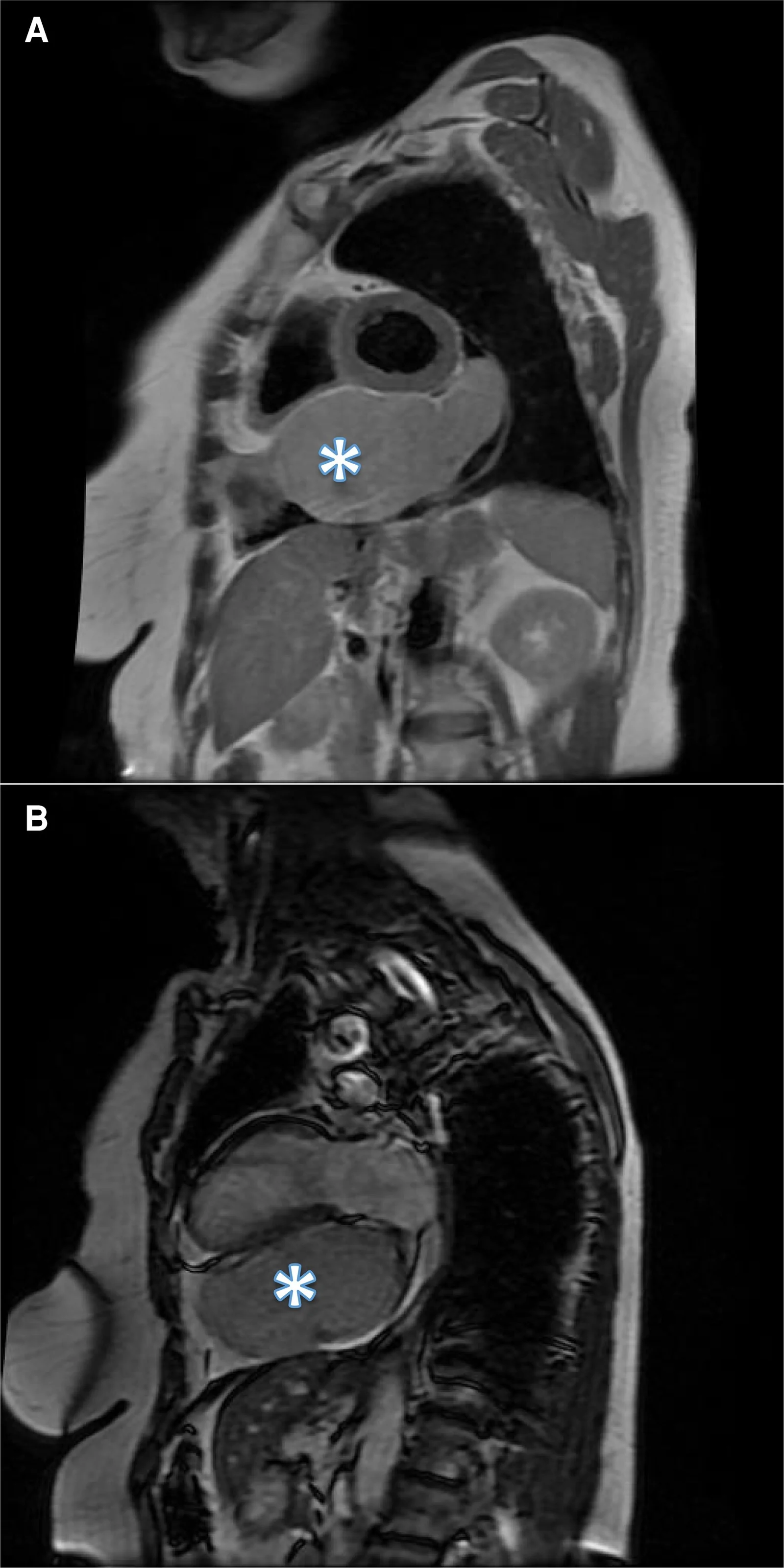
Cardiac magnetic resonance imaging demonstrating a large heterogeneous pericardial mass with internal septations and moderate pericardial effusion. (*A*) Coronal view. (*B*) Sagittal view.

Coronary angiography ruled out obstructive coronary lesions but revealed an anomalous vessel originating from the posterior interventricular artery, possibly supplying the tumour (*Figure 3*).

**Figure 3:**
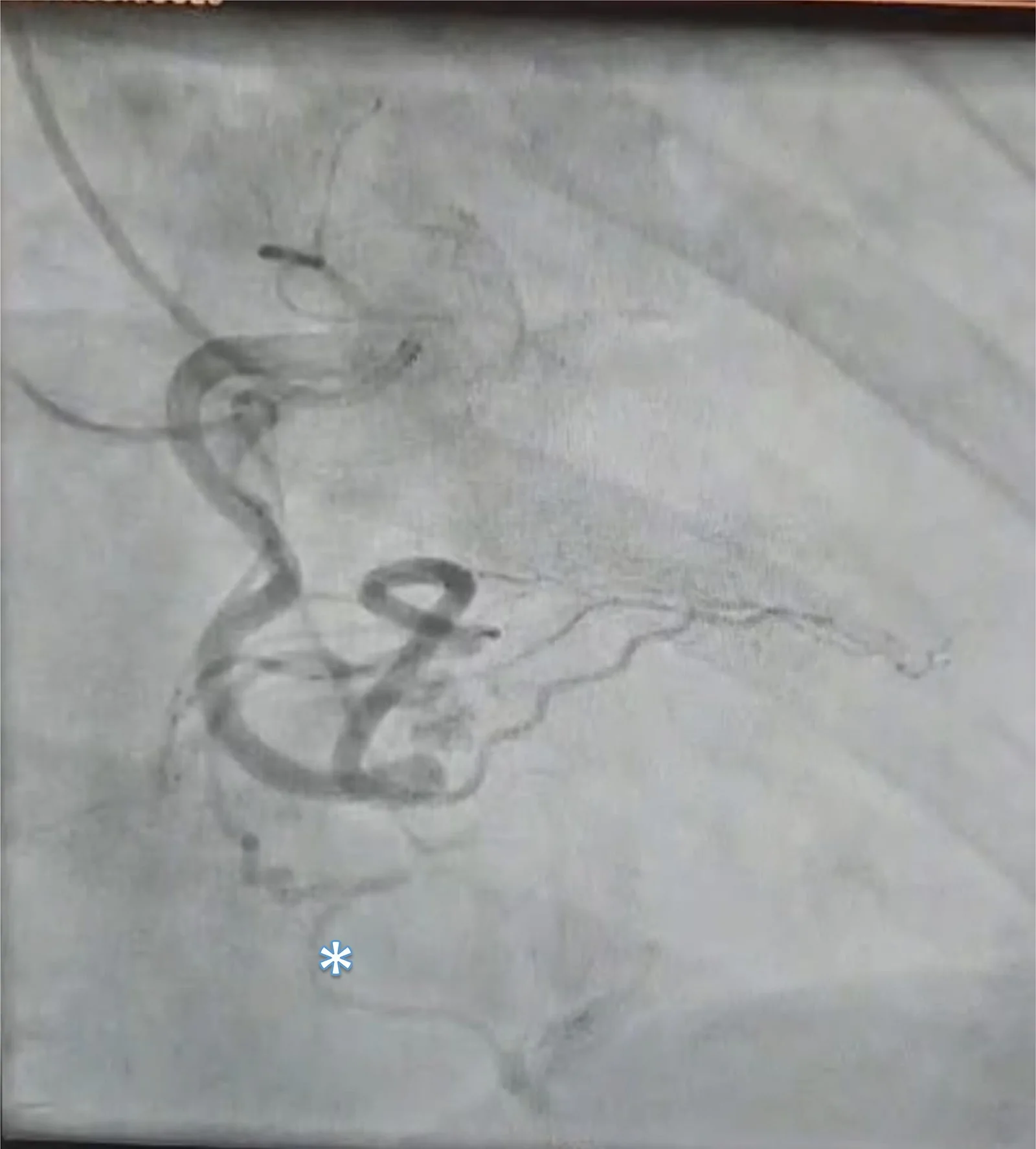
Coronary angiography showing an anomalous vessel originating from the posterior interventricular artery and possibly supplying the tumour.

Surgery was performed under general anaesthesia, with a median sternotomy. Central cannulation for cardiopulmonary bypass was established using ascending aortic arterial cannulation and bicaval venous cannulation. Total cardiopulmonary bypass time was 31 min. Aortic cross-clamping was not required, and the procedure was performed on the beating heart without cardioplegic arrest.

After the establishment of cardiopulmonary bypass, a lobulated mass adhered to the diaphragmatic surface of the heart was completely resected (*Figure 4*). The tumour originated from the epicardium of the right atrium, extending to the interventricular septum, inferior vena cava, and the inferior wall of the left and right ventricles. The posterior interventricular artery, responsible for nourishing the tumour, was ligated without causing damage to the main coronary circulation. Reconstruction of the inferior vena cava, right atrium, and right ventricle was performed using simple sutures.

**Figure 4:**
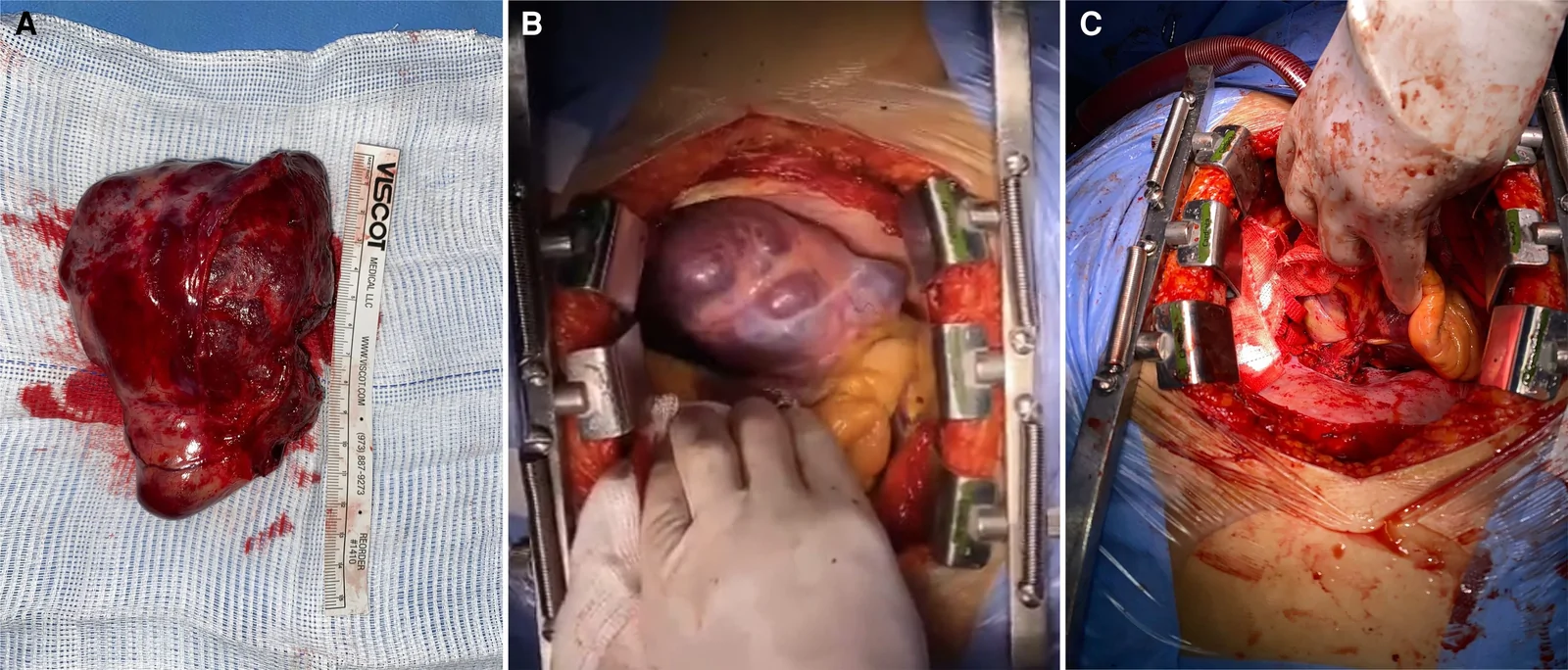
Intraoperative and macroscopic aspects of the cardiac mass. (*A*) Resected surgical specimen. (*B*) Intraoperative view demonstrating the tumour before resection. (*C*) Surgical field after complete tumour excision.

Histopathological examination confirmed the diagnosis of a cavernous haemangioma of cardiac origin (*Figure 5*). The patient had a satisfactory postoperative course, being discharged from the hospital 3 days after surgery. One month after the procedure, echocardiography and MRI showed no signs of residual mass or functional compromise of the heart. The patient was considered cured and referred for annual follow-up.

**Figure 5:**
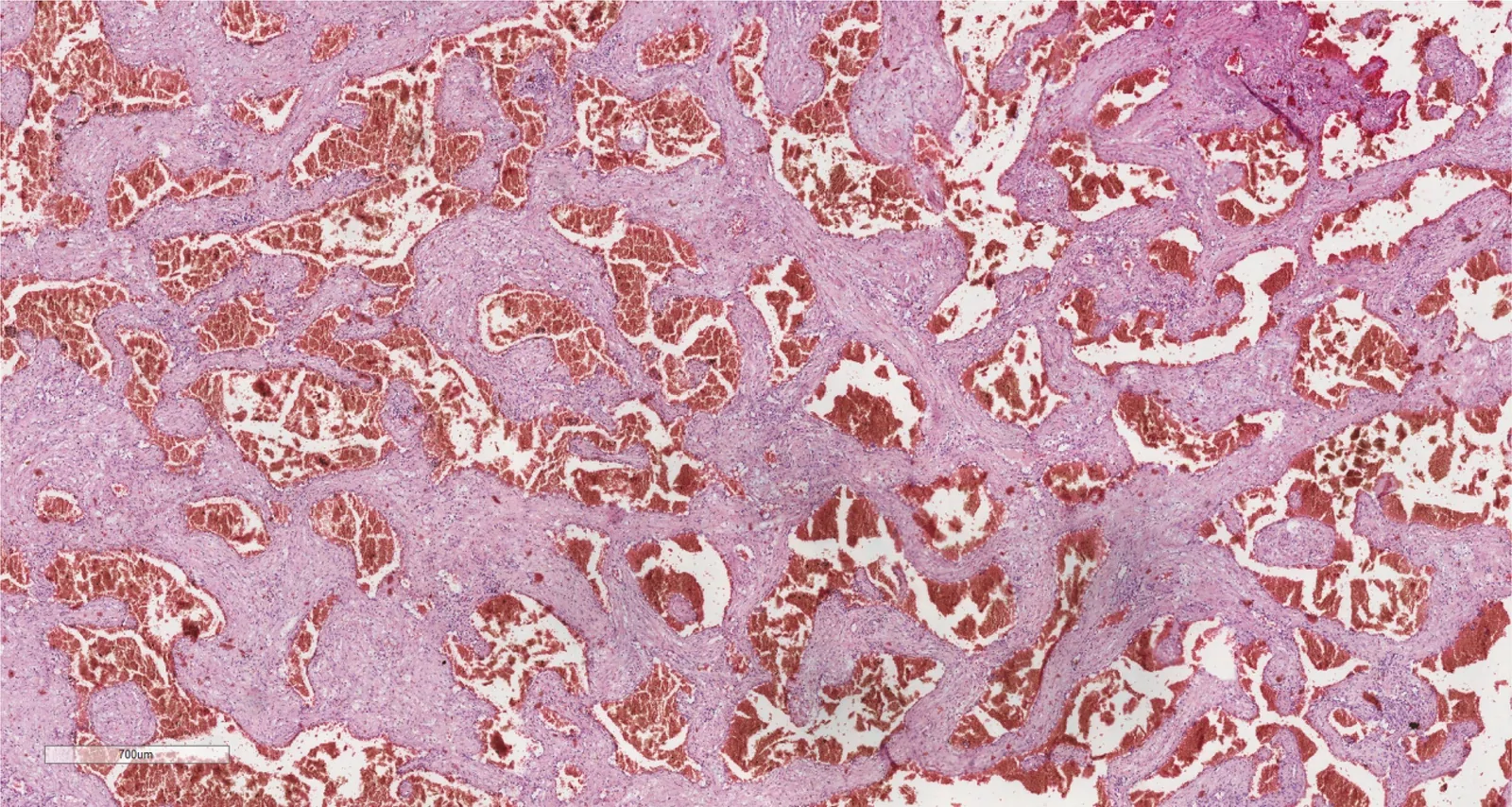
Histopathological examination demonstrating cavernous haemangioma of cardiac origin.

## Discussion

Cardiac haemangiomas are exceedingly rare, with an incidence of less than 0.02% among primary cardiac tumors.^3,7^ Despite their rarity, they present significant clinical and diagnostic challenges. These tumours may remain clinically silent and are frequently identified incidentally during imaging or autopsies. However, as seen in this case, they can become symptomatic due to their size or location, causing compression of adjacent cardiac structures and leading to clinical manifestations such as arrhythmias, heart failure, or haemodynamic compromise.^4,5^ Cavernous haemangiomas, the most frequently reported subtype, exhibit expansive growth patterns and significant vascularity, which may exacerbate these clinical challenges.^3,6^

The differential diagnosis of cardiac haemangiomas includes other benign cardiac tumours, such as myxomas and lipomas, as well as intracardiac thrombi. Differentiating these entities can be challenging due to overlapping clinical and imaging features. Echocardiography is an invaluable initial diagnostic tool, offering real-time information on tumor size, location, and haemodynamic impact. However, echocardiography alone may not provide sufficient detail for definitive diagnosis. Advanced imaging modalities, particularly contrast-enhanced MRI and computed tomography, are crucial for characterizing the tumour’s vascularity, extent, and involvement of surrounding structures.^8–10^ In this case, MRI was instrumental in confirming the tumour’s dimensions, internal characteristics, and vascular supply, enabling precise surgical planning.

Surgical resection remains the cornerstone of treatment for cardiac haemangiomas. Complete excision significantly reduces the risk of recurrence and ensures a favourable prognosis.^10^ In this case, meticulous surgical planning allowed for the complete removal of the tumour, including ligation of its vascular supply, without compromising the integrity of major cardiac structures. The use of central cardiopulmonary bypass without aortic cross-clamping allowed safe resection on the beating heart, avoiding cardioplegic arrest while maintaining adequate exposure and haemodynamic control. The patient’s uneventful postoperative recovery and absence of residual tumour on follow-up imaging further underscore the importance of complete resection. However, incomplete excision poses a substantial risk of recurrence, highlighting the need for precise surgical technique and long-term postoperative monitoring.^7,9^

Imaging also plays a critical role in postoperative management. Periodic follow-up with echocardiography and MRI is essential to monitor for potential recurrence or late complications. Although rare, recurrence has been reported in cases of incomplete resection or residual tumor.^3,9^ Additionally, advanced imaging techniques are invaluable in evaluating functional outcomes and ensuring that no significant structural or haemodynamic abnormalities persist.^8,10^

This case illustrates the critical importance of a multidisciplinary approach to managing cardiac haemangiomas. Collaboration between cardiologists, radiologists, and surgeons is essential for accurate diagnosis, effective surgical intervention, and comprehensive postoperative care. The use of advanced imaging modalities not only facilitates precise preoperative assessment but also enhances long-term surveillance, ensuring optimal patient outcomes.^3,10^

## Conclusion

Although rare, cardiac haemangiomas should be considered in the differential diagnosis of benign cardiac tumours, especially in cases of large masses with evident vascular characteristics. The use of advanced imaging technologies is crucial for diagnosis and surgical planning. Complete resection of the tumour, as demonstrated in this case, is the treatment of choice and results in a favourable prognosis, with a low rate of complications and recurrence. Long-term follow-up is recommended to ensure the absence of recurrence.

## Lead author biography


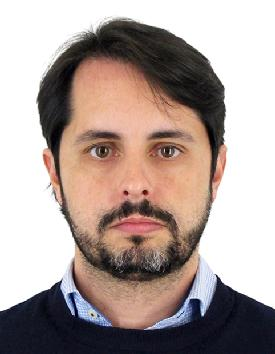


Henrique Madureira da Rocha Coutinho is a cardiovascular surgeon at Pedro Ernesto University Hospital (UERJ) and Serra dos Órgãos University Center (UNIFESO), Rio de Janeiro, Brazil. He serves as Coordinator of the Cardiovascular Surgery Service and Residency Program and is actively involved in academic training and clinical research. He obtained his medical degree in 2011, completed general and cardiovascular surgery training in Brazil, and holds a Master’s degree, currently pursuing a PhD at UERJ. His academic interests include cardiac surgery, perioperative myocardial protection, rare cardiac pathologies, and clinical outcomes research. He is also a peer reviewer for international cardiology journals.

## Data Availability

The data underlying this article are available within the article. Additional data are available from the corresponding author upon reasonable request.
